# An appraisal of students' awareness of "self-reflection" in a first-year pathology course of undergraduate medical/dental education

**DOI:** 10.1186/1472-6920-11-67

**Published:** 2011-09-23

**Authors:** Rani Kanthan, Jenna-Lynn B Senger

**Affiliations:** 1 Department of Pathology and Laboratory Medicine, College of Medicine, University of Saskatchewan, Saskatoon Saskatchewan, Canada

## Abstract

**Background:**

Self-reflection and reflective practice are increasingly considered as essential attributes of competent professionals functioning in complex and ever-changing healthcare systems of the 21^st ^century. The aim of this study was to determine the extent of students' awareness and understanding of the reflective process and the meaning of 'self-reflection' within the contextual framework of their learning environment in the first-year of their medical/dental education. We endorse that the introduction of such explicit educational tasks at this early stage enhances and promotes students' awareness, understanding, and proficiency of this skill in their continuing life-long health professional learning.

**Methods:**

Over two years, students registered in first-year pathology at the University of Saskatchewan were introduced to a self-reflection assignment which comprised in the submission of a one-page reflective document to a template of reflective questions provided in the given context of their learning environment. This was a mandatory but ungraded component at the midterm and final examinations. These documents were individually analyzed and thematically categorized to a "5 levels-of-reflection-awareness" scale using a specially-designed rubric based on the accepted major theories of reflection that included students' identification of: 1) personal abilities, 2) personal learning styles 3) relationships between course material and student history 4) emotional responses and 5) future applications.

**Results:**

410 self-reflection documents were analyzed. The student self-awareness on personal learning style (72.7% level 3+) and course content (55.2% level 3+) were well-reflected. Reflections at a level 1 awareness included identification of a) specific teaching strategies utilized to enhance learning (58.4%), b) personal strengths/weaknesses (53%), and c) emotional responses, values, and beliefs (71.5%). Students' abilities to connect information to life experiences and to future events with understanding were more evenly distributed across all 5 levels of reflection-awareness.

**Conclusions:**

Exposure to self-reflection assignments in the early years of undergraduate medical education increases student awareness and promotes the creation of personal meaning of one's reactions, values, and premises in the context of student learning environments. Early introduction with repetition to such cognitive processes as practice tools increases engagement in reflection that may facilitate proficiency in mastering this competency leading to the creation of future reflective health professionals.

## Background

As Confucius said, "By three methods may we learn wisdom: first, by reflection, which is the noblest; second, by imitation, which is the easiest; and third, by experience, which is the most bitter". Dating back to ancient Greece, the 'noble' process of reflection has developed into an effective teaching/learning tool capable of enhancing education, decision-making, critical thinking, and self-concept. Innovation, problem-solving, autonomy, and critical thinking are all necessary skills in healthcare professionals [1]. When performed correctly, reflective writing develops these indispensable attributes, encouraging the writer to create meaning from events that have transpired to guide future choices [2]. As with any skill, the ability to productively reflect requires practice; therefore, implementation of activities exercising this skill early in one's healthcare career should increase student engagement and prepare doctors/dentists-in-training to be competent in these skills in their respective chosen profession.

Now, over 2,500 years after the time of Confucius, the young population is reflecting more than ever. Though not in an academic setting, social media networks such as facebook, myspace and twitter are based on users recording their thoughts and actions, and commenting on what others have written. Despite what is often a daily ritual of recording and discussing (ie "social reflection"), in an academic context, students often struggle with the reflective process. Studies have shown low-levels of reflection in residents and medical students, suggesting that the cognitive processes required to enhance learning through reflection may not be intuitive and suggests a role for active educational interventions [3]. A true reflective process is multidimensional including recognition, implementation and application with a view to increase the depth of understanding. Such increases may involve an evaluation of performance, consideration of events causing these outcomes, and affective and behavioral reactions leading to single-, double- and triple-loop learning [4,5]. The concept of reflection, though widely mentioned in medical education literature, often describes similar processes under different terms. The main approaches to reflection in medical education are threefold: a) reflection for learning, b) reflection to develop a therapeutic relationship and c) reflection to develop professional practice. Reflection can also be considered primarily as a self-regulated learning activity [5]. In contrast to "social reflection", accurate definition of "academic reflection" is recently articulated as twelve tips for teaching reflection at all levels of medical education by Aronson. These twelve tips are: "1) define reflection 2) decide on learning goals for the reflective exercise, 3) choose an appropriate instructional method for the reflection, 4) decide whether you will use a structured or unstructured approach and create a prompt, 5) make a plan for dealing with ethical or emotional concerns, 6) create a mechanism to follow-up on learners' plans, 7) create a conducive learning environment, 8) teach learners about reflection before asking them to do it 9) provide feedback and follow-up, 10) assesses the reflection, 11) make this exercise part of the larger curriculum to encourage reflection, and 12) reflect on the process of teaching reflection" [6]. With these criteria taken into account to maximize reflection effectiveness, the ultimate goal is the creation of a mindful practitioner, with new initiatives encountered by students acting as building blocks towards this end [3]. As such, identification of specific facets that make up an effective "self-reflection" is important. The awareness of these skills in first-year medical/dental students will facilitate and guide the development of self-reflective exercises that can be specially tailored to suit students' needs to create a firm foundation towards this final objective.

In this research paper we share our experience of an explicit initiative to implement a self-reflection assignment in the first-year pathology Med 102 course. We were interested in determining the extent of students' awareness and understanding of the reflective process and meaning of 'self-reflection' within their learning environment in this early stage of their education.

## Methods

In the academic years 2007-2008, a total of 95 students (66 medical and 29 dental) and in 2008-2009 a total of 110 students (84 medical and 26 dental) at the University of Saskatchewan were introduced to a self-reflection assignment as part of their pathology course. Students' age ranged from 19 to 34 (mean 23 years) and past educational history ranged from two years of post-secondary to a Master's degree. This wide age range is due to the fact that students in this province are eligible to apply following 2 completed university years. Successful applicants in this category are usually a minority comprising 10-15% of the overall selected candidates. The College of Medicine at the University of Saskatchewan follows a ***CASE-***based (***C***ooperative, ***A***ctive, ***S***elf-directed, and ***E***xperiential learning) integrated four-year curriculum. A balanced composite curricular design including facets of horizontal, vertical and diagonal integration of pathology teaching in the first three years is adopted as the best practice plan for the instruction of pathology within this integrated medical curriculum [7]. The first year courses are attended by both medical and dental students thus promoting opportunities for inter-professional exchanges. The first year pathology, MED 102, is related to the teaching of general pathological concepts serving as a foundation to the upcoming systemic pathology taught in second (MED 202) and third (MED 302) year courses. The MED 102 first year pathology course provides students with an overview of basic concepts and principles of general pathological conditions as applicable to real life practices of medicine and dentistry with horizontal integration to the introductory first year Form and Functions course (a comprehensive vertically integrated histology, embryology, anatomy and physiology course that is run as modular units). The MED 202 (second year) and 302 (third year) courses are horizontally integrated with the teachings of the various scheduled clinical systems with ongoing vertical and diagonal integration of pathology within the matrix of the undergraduate curriculum [7]. Further, in keeping with the ***CASE ***curriculum, students were introduced to the new pathological concepts through active learning strategies including in-class jigsaw learning, pre-assigned reading assignments with presentations, specially designed pathology games, poster creations, impromptu quizzes, Turning Point questions with clicker responses, and case explorations with in-class debates that complemented the traditional formal lecture sessions. All first-year students are aware of the concepts of multiple intelligences with preferred learning styles that serve as the underlying structural framework for the ***CASE ***curriculum model.

Following an in-class discussion of the proposed self reflective assignment, in the context of their learning experiences, students were provided with a reflective questionnaire template as a practice assignment (Appendix 1). Students were requested to submit a one-page (500 word) reflective document of their learning experiences as an ungraded (pass/fail course) but compulsory component of the midterm and final examinations. These documents were collected over two academic years amongst two cohorts of students (2007-2008; 2008-2009). The documents were randomly analyzed through a blind evaluation process by two investigators that each read and evaluated the entire set.

Validated instruments are available in medicine to assess critical thinking regarding well defined clinical problems. The Gronigen Reflection Ability Scale (GRAS) is a one dimensional practical measurement instrument that contributes to valid inferences about the personal reflection ability of medical students and doctors, both at individual and group level. The main emphasis of this scale is on empathetic reflection and reflective communication [8]. However, in our study we wished to evaluate and measure student's awareness and understanding of self- reflection in the context of their learning environment. These student perceptions were collected as narrative documents based on a suggested template. The student's qualitative comments were categorized vertically into 5 levels of understanding with horizontal integration into seven categories and developed as a rubric (Table 1). Such categorization pooled common and similar student comments to attain numerical percentages for comparative discussion.

**Table 1:** Reflective Rubric

Category	**The student identifies**:	Level 5	Level 4	Level 3	Level 2	Level 1
**A**	**Significant events from course that led to reflection**	Skillfully details precise accounts of important events	Several events are accurately logged in detail	Attempts to outline in some detail one activity	Generally describes the entire scope of the class without specific detail	Does not select and summarize activities that have transpired

**B**	**Information/Skills acquired from course**	Demonstrates in depth understanding of skills	Demonstrates a thoughtful awareness of skills	Demonstrates a rudimentary awareness of some skills practiced	Demonstrates a limited awareness of few skills/strategies practiced throughout the year	Demonstrates little to no awareness of skills targeted

**C**	**Personal abilities**	Demonstrates ability to list all strengths and weaknesses and provide a plan for amelioration	Demonstrates ability to list some strengths and weaknesses	Demonstrates ability to list at least one strength and one weakness	Demonstrates ability to recognize personal strengths but not weakness	Does not demonstrate ability to see personal strengths or weaknesses

**D**	**Personal learning style**	A concise recognition of learning requirements and ways to facilitate future learning is identified	Recognizes a preferred learning style and compares it to teaching style employed in class	Recognizes preferred learning style	Indicates a preference of one activity from class over another	No evidence of recognition is made

**E**	**Connections between the course and past life experiences**	Connections are explored in great precision and detail	Connections are made and casually discussed	Connections are recognized but are ineffective	Limited connections to nonspecific, theoretical aspects of life are discussed	No evidence of connections are made

**F**	**Oneself in relation to the situation (emotions, values, beliefs, etc.)**	Writing reveals a well-developed emotional and personalized relationship to the specific context, enhanced with specific examples	Writing reveals a strong connection between the event and the writer accompanied by few general reflections	Both general and personal insight are present, further exploration is required	Generalizations used to explore topics of discussion with 1-2 references to self	Employs no 'self' into writing; uses only obvious statements without contextual references

**G**	**Future applications in a broadened spectrum**	Explicit details of precise aspects of course load and relates them to specific anticipatory future events	Recognition of few precise important facets of course load expanded to broad future goals	Generalized discussion of possible future implications is discussed is some detail	Future applications of an aspect of the course are mentioned in passing without detail	No future applications of class materials are discussed

Reflections were categorized on a "5 levels of reflection-awareness" scale using a specially designed rubric based on the accepted major theories of reflection, with level 1 representing the poorest understanding of reflection and level 5 representing greater understanding (Table 1). Documents were categorized based on the reflection-awareness of: 1) personal abilities, such as strengths and weaknesses 2) personal learning styles 3) relationships between course material and the students' personal histories 4) emotional responses and relations to personal values, beliefs, and sense of self and 5) future applications of the material/experience being discussed at both a personal and professional level. The levels of reflection-awareness (1-5) analyzed in these documents were also vertically categorized in the following dominant themes consistent with existing literature on reflection:

**Category A: **Significant events from course that led to reflection

**Category B: **Information/Skills acquired from the course

**Category C: **Personal abilities

**Category D: **Personal learning style

**Category E: **Connections between the course and past life experiences

**Category F: **Oneself in relation to the situation (emotions, values, beliefs, etc.)

**Category G: **Future applications in a broadened spectrum

It is important to recognize that the 'levels of reflective-awareness' does not represent a strictly linear scale but rather attempts to classify five different levels of students awareness and understanding of the self- reflective process within the context of their learning environment in this early stage of their education.

Following independent analysis by each investigator the results in agreement were pooled. The disagreed results were discussed and reappraised to reach a mutually acceptable consensus opinion. As assessment of reflection is difficult to be linked to explicit educational goals of learning and understanding [5], a combination of such qualitative analysis with quantitative numerical categorization was specifically used in this study based on past experiences [7].

This study was conducted with ethics approval from the University of Saskatchewan Behavioral Research Ethics Review Committee.

## Results and Discussion

**Figure 1:**
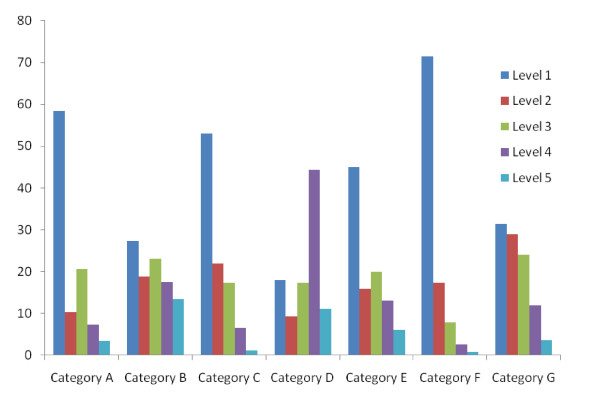
**This is a graphic representation of the qualitative analysis of the students' self-reflective documents**. The y-axis shows the percentage of student responses in the various levels of reflection to the various categories as listed on the x-axis. Level 1 indicates a limited reflection-awareness while level 5 represents a well-reflected response. The categories on the x-axis are as follows: **Category A: **Significant events from course that led to reflection. **Category B: **Information/Skills acquired from course. **Category C: **Personal abilities. **Category D: **Personal learning style. **Category E: **Connections between the course and past life experiences. **Category F: **Oneself in relation to the situation (emotions, values, beliefs, etc.).

Over the course of two academic years a total of 410 self-reflection documents were collected and analyzed from the two student cohorts. A sample of 'student comments' from each category (A-G) and level (1-5) is provided in Appendix 2. Students demonstrated a strong ability to outline their personal learning style (Category D: 17.3% level 5- "*I learn best through multiple modalities where I can make interconnections"*, 44.3% level 4-"*I am an extreme multiple-modality learner"*, 11.2% level 3-" *I find that I am a visual learner"*). This may be attributed to the template that specifically prompted students to include this in their discussion. The majority of the students included some detailed mention of the course content (Category B: 13.4% level 5 -" *I need this general pathology to understand the mechanisms causing systemic oral diseases and the body's reactions to them such as inflammation encountered in gingivitis "*, 17.5% level 4 -"*I enjoyed the sessions on neoplasia and atherosclerosis because of the value of this information. These illnesses are the two leading causes of death worldwide making learning how they develop more interesting"*, 23.1% level 3 - "*In the second portion of this course we focused on neoplasia, atherosclerosis, infectious disease, and environmental pathology"*). In this context, many (> 85%) submitted a 'log-book', or an account of some of the major topics covered, without providing any evidence of deeper understanding with critical thinking. As seen in Figure 1, Categories E ("Connections between the course and past life experiences") and F ("Students identified oneself in relation to the situation"), the majority of students were at a level 1-2, indicating that they struggled to connect information from the course to 'the self' and thereby failed to personalize their learning. Reflections at level 1 awareness included a) the identification of specific teaching strategies utilized to enhance learning (Category A: 58.4% - "*I think that I have learned many things throughout this course"*), b) identification of personal abilities (Category C: 53% *Did not mention any strengths and weaknesses*:), and c) the relation of emotional responses, values, and beliefs to the material being covered (Category F: 71.5% - "*I enjoy topics more as I collect more knowledge about them"*). Students' abilities to connect information to future events (Category G) were more evenly distributed across all 5 levels of awareness (Figure 1).

Self-directed learning such as that used by lifelong learners requires "proactive rather than reactive thought" [1]. This novel thought pattern may result from a myriad of experiences culminating together and strengthened with input from personal emotions and ideals. The word 'experience', originates from Latin for 'trial' or 'experiment', indicating a part of education that must include trial-and-error learning [9]. Nearly 100 years ago, in 1933, John Dewey was the first to write about the value of reflection, suggesting there is more to learning than simply varied experiences [10]. Since Dewey, numerous researchers have attempted to outline steps to the reflective process and pinpoint the precise benefits of this action. Theories of many influential thinkers such as Dewey, Boud, Schön, Moon, Mezirow, and Kolb share commonalities that together may provide the fundamentals of understanding reflection. In 1985 Boud et al. conclude that structured reflection is a key element in learning and proposed a reflective process whereby one must: a) mentally return to the experience, b) identify/attend to feelings that arise in response to these events with recognition of self-held 'truths' barring full understanding, and c) re-evaluate the experience through association, integration, validation and appropriation [11]. Information gained from such a process can then be used to guide future decisions and choices. Varied structures of this reflective process are echoed in the theories of McCarthy [12] and Kolb [13]. Such iterative processes often begin with (i) the identification of a problem which is resolved (ii) the collection and analysis of pertinent information/emotions, and (iii) the incorporation of the same into future actions. The critical step of this process is the second (ii); the investigation of the positive/negative emotions triggered by an event tempered by the underlying beliefs and self-values that determines one's decision and choice [14]. Zull states "Even if we experience something that has happened to us before, it is hard to make meaning of it unless it engages our emotions" [15]. Only a few students in our study were able to effectively connect information to personal experiences (Category E Level 4-" *I found that having a personal experience with cancer in my family increased by interest in the lectures dramatically"*) and self-values (Category F Level 5- "*I was very interested in the atherosclerosis and obesity section. I come from a population that has a high rate of obesity, diabetes, and many other diseases so I feel that as a future health professional, it is one of my responsibilities to try and promote healthy choices. Improvements will be made with proper awareness and education as well as with positive role models in the community like health care professionals with a background in these conditions."*), congruent with models of reflections by Mezirow [16], and Hatton/Smith [17] that values 'critical analysis' at the highest level of reflection. This may be attributed to lack of previous exposure to such critical thinking tasks, perhaps directly related to inexperience as approximately 45% of the students had not completed a four year undergraduate university degree prior to their acceptance into medical/dental school. Students' written language competencies could also have a significant bearing on the overall assessment of these narratives, in terms of the student's ability of effective written communication skills. However, for the purposes of this study it was assumed that all students possess the necessary written communication skills needed for this assignment as they had at least 2 years of university education and many had 3-6 years of university experience.

Schön suggests three types of reflection that may occur: 1) reflection-on-action (reflection post-event), 2) reflection-in-action (reflection during the time of action), and 3) knowing-in-action (intuitive knowing established from repetitive practice) [18]. Each of these reflective processes must include the various elements as discussed above. Through 'reflection-on-action' one may develop the ability to choose correctly as a 'knowing-in-action' reflection. In our study, the self-reflection assignment was undertaken as part of the structured course, focusing on events of the past and concurrent activities as stimuli for learning, both within the classroom and in the extended learning environment. In our specially-designed "5-levels of reflective-awareness" rubric (Table 1): 1) categories A and B evaluated students' abilities to 'reflect-on-action', which is also the first step in Schön's reflective process [18]; 2)categories C, E, and F assessed the second criteria, reflection-in-action, as students identified aspects of themselves within context of the situation as it occurred; and 3) categories D and G appraised the ability to 'know-in-action', by in-the-moment identification of one's abilities with future directions.

Reflection and reflective practice are essential attributes for competent healthcare professionals that have to function in complex, changing healthcare systems. Formal requirements for practitioners to provide evidence of reflective practice is becoming mandatory in many of the licensing and re-validation processes of the governing bodies such as the College of Family Physicians of Canada, Royal College of Physicians and Surgeons of Canada, and General Medical Council UK [19]. Therefore, it is imperative that early and repetitive practice to acquire competency in this skill is explicitly included in the curriculum in the early years of undergraduate medical education.

In its entirety, reflection as a cognitive and affective process or activity is aimed at broadening and deepening one's understanding of the choices available through active engagement by the individual that may be triggered by an unusual experience and involves examining one's responses, beliefs, and premises to this situation resulting in an integration of the new understanding into one's personal experience. Thus, this reflection may be influenced by the situation itself, or by factors related to the individual and/or the larger environment. The process of reflection begins with the identification of a problem and a deliberate decision to seek a mindful solution; yet, the process of reflection does not always have a defined beginning and end, leading to further reflection with continued deeper understanding [14]. In our study, this assignment embodied an example of reflective practice that required students to act and think professionally as an integral component of in-course learning, facilitating knowledge transfer from in-class to professional practice [19]. Sobral demonstrated that an increased effort at reflection is associated with a more positive/meaningful learning experience with greater enjoyment of studies, and higher scores in diagnostic thinking and problem-solving. Increased reflection was also associated with greater perceived learner autonomy. Such 'reflection-in-learning' facilitated by educational interventions such as self-reflective assignments could induce readiness for stronger self-regulation of learning with enhanced diagnostic thinking ability [20]. The changing, intricate healthcare system requires clinicians to continually refresh and update their knowledge and skills to solve complex patient problems [19]. These traits, if specifically targeted by repetitive exposure to reflective practice assignments commencing in the early years of undergraduate medical/dental students' career, may result in an enhanced education with personal/professional effectiveness of critical thinking skills [14]. Through an increased awareness, understanding and recognition of their own personal learning styles, abilities, responses, and beliefs, medical/dental students may develop a readiness for application with new perspectives and a commitment to action through effective and enhanced critical thinking skills [20].

Though the broad concept of self-reflection has been thoroughly outlined in the literature, specific references to the stage of life at which such reflection should begin are sparse. The beginning of one's professional training is crucial for the development of a future professional identity [21]. To fully develop capabilities needed to succeed in any profession, exposure and ongoing reflection are necessary [22]. Students often enter medicine/dentistry with certain expectations; self-regulation enables the development of more accurate perceptions with greater flexibility and creativity [14]. Encouraging students to begin their reflective practices during the early years of medical/dental school establishes both a foundation for personal and professional development with a set of rudimentary critical thinking skills which may be expanded and perfected through the remainder of their professional career.

In their reflective documents, several of the students commented on the perceived usefulness of this activity and the importance that they believed reflective practice would have in their future. The majority of students (> 75%) who reflected on the learning process responded favorably to this activity, commenting "*This assignment has made me more self-aware of the best way that I learn. This makes me want to try harder to find the learning methods that work best for me so that I can maximize how much I am able to learn"*. Students felt this 'reflection-on-action' contributed to their learning, commenting "*There is a unique component in this course- this document. After finishing a course there is usually no time to look back at what I learned - not only at the material, but why I learned it and how I can use the knowledge. Because I will care for real patients someday, I know that I need to retain the knowledge and learn the most effective way to gain new insight; this effective learning activity teaches me to do just that"*. A few students (< 10%) however, did state they felt the assignment was a "waste of time" and that this was an 'assessment' that was 'highly prone to subjectivity'. This latter cohort of students had decreased awareness in many of the levels of understanding in comparison to students that valued this particular assignment as an educational growth activity. This suggests that increased awareness with enhanced reflective abilities may provide a deeper understanding of the importance of reflection and reflective practice in the educational development of healthcare professionals. These findings, however, are limited as they are an educational intervention snapshot of the students' reflections in a single course of their undergraduate education. Additionally, these findings are dependent entirely on the individual perception of the given cohort of students.

In summary, we recommend that such reflective processes be incorporated as part of a continuum in the overall curriculum design of the undergraduate education of healthcare professionals. It is well-known that in the student world, assessment drives learning. However, prioritizing self-reflection as an assessment tool is a daunting task, as one's depth of reflection is a) difficult to evaluate objectively, b) time-consuming to assess, and c) challenging to standardize. Nevertheless, with effective feedback of such educational interventions as part of formative assessment, learners can be provided with information to enhance their learning/practice with critical thinking skills, to result in affecting positive change [23].

## Conclusion

Though reflection has existed for many millennia and continues in the current digital era as "social reflection" practices, its value in enhancing higher education is only beginning to be explored in the current literature. The process of reflecting and its benefits in enhancing learning and personal growth are emerging in the research domain as an effective teaching/learning tool that may be implemented at any level of education. The introduction of self-reflection early in undergraduate medical/dental education promotes the creation of personal meaning of one's reactions, values, and premises in the context of student learning environments. Early introduction to such cognitive processes with repetition serve as practice tools to develop and become proficient in mastering this competency resulting in the creation of future reflective health professionals.

## Competing interests

All authors declare that they have no financial or non-financial competing interests. This study was conducted with ethics approval from the University of Saskatchewan Behavioral Research Ethics Review Committee.

## Authors' contributions

RK is the corresponding, and first author of this manuscript. JLS is a medical student who has contributed to the acquisition, analysis, and interpretation of data. Both authors have read and approved the final manuscript and assume full responsibility for its contents.

## Appendix 1

### Suggested Template for Self-Reflective Assignment in Med 102

The self reflective document will be a typed one page document of approx [300-500] words.

This reflective piece will be a self testimonial for you to pause, reflect and share on the what, who, why, where, when, and how's of your learning experience in this course.

Some guided questions to help you frame your thoughts are as follows:

a) What have I learnt? How? Why is it valuable?

b) When, where have I learned? Circumstances/conditions/peers.?

c) Do I know what kind of learner I am?

d) What difference has this learning made? What is the contribution of this learning to my pool of knowledge?

e) How does this experience fit into my larger personal goals and achievement of being a medical doctor/dentist?

f) What else would I like to learn? Why? And how will I go about learning it?

## Appendix 2

### A sample of student comments for each category (A-G) and reflection-awareness level (1-5)

#### A. Significant Events from Course that Led to Reflection

##### Level 1

Does not select and summarize activities that have transpired

- "*I have never taken a course in pathology before, and find that it is challenging and quite different from anything I have learned before."*

- *"I think that I have learned many things throughout this course."*

##### Level 2

Generally describes the entire scope of the class without specific detail

- "*This is the first course in which we are not just learning about normal physiology but starting to look at the disease processes that deviate from the normal"*

##### Level 3

Attempts to outline in some detail one activity

- "*The matching assignment helped to foster friendships between the dental and medical students by promoting interaction as helping to integrate knowledge in an effective way - discussion."*

##### Level 4

Several events are accurately logged in detail

- "*I learned from the class lectures and clicker questions about amyloidosis, inflammation, and the different types of cell death. I have learnt the most from the interactive activities such as making a thrombus out of "fibrin" and platelets. That is a learning experience that will stick with me much longer than any amount of time in didactic lectures."*

##### Level 5

Skillfully details precise accounts of important events

- "*I I found the most recent lecture on hemorrhage, thrombosis, embolism and infarction intriguing. There was a clear structure and concise presentation making it easy to follow. The demonstration of thrombosis with the toilet-paper rolls and candy involved a more complicated concept that would have otherwise been hard to grasp and the beta-pleated sheet exercise(structure of amyloid) was useful in reminding me of my past biochemistry class."*

#### B. Information/Skills Acquired from Course

##### Level 1

Demonstrates little to no awareness of skills targeted

- "*Over the second half of this semester we have learned about some pathology topics that were interesting."*

##### Level 2

Demonstrates a limited awareness of few skills/strategies practiced throughout the year

- "*I marvel at the organizational complexities of the human body as I learn about a body system, defense mechanism or developmental sequence"*

##### Level 3

Demonstrates a rudimentary awareness of some skills practiced

- "*In the second portion of this course we focused on neoplasia, atherosclerosis, infectious disease, and environmental pathology."*

##### Level 4

Demonstrates a thoughtful awareness of skills

- "*I enjoyed the sessions on neoplasia and atherosclerosis because of the value of this information. These illnesses are the two leading causes of death worldwide making learning how they develop more interesting."*

##### Level 5

Demonstrates in depth understanding of skills

- "*I need this general pathology to understand the mechanisms causing systemic oral diseases and the body's reactions to them such as inflammation encountered in gingivitis. The information of this class can be combined with the physiology and pharmacology classes to study drug interactions with the various diseases we are learning."*

#### C. Personal Abilities

##### Level 1

Does not demonstrate ability to see personal strengths or weaknesses

- Student reflections that did not mention any strengths or weaknesses were grouped into this category.

##### Level 2

Demonstrates ability to recognize personal strengths but not weakness

- "*I think due to my new study habits I am actually retaining information beyond the day of the test, which was a major goal coming into medical school."*

- "*My abilities to not only memorize the material and learn and retain concepts have improved greatly."*

##### Level 3

Demonstrates ability to list at least one strength and one weakness

- "*Midway through the semester I was beginning to feel down about the limited time I had for a vast amount of work; however, I buckled down and did better than I hoped on the midterm which restored some of my confidence."*

- "*I need to work on spreading out my learning throughout the course. I work better in shorter pockets of time."*

##### Level 4

Demonstrates ability to list some strengths and weaknesses

   - (From one entry):

○ "*One thing I still struggle with is memorization. We take a huge course load that covers an enormous volume of material and I struggle to remember it all"*

○ "*I must admit I have developed a poor habit of wasting much of my time spent in lectures. I find myself leaving lectures and wondering if I actually retained any of the information that was presented"*

○ "*I found that by listening for pure interest sake, I retain more information than by furiously taking notes"*

##### Level 5

Demonstrates ability to list all strengths and weaknesses and provide a plan for amelioration

- "*I had a great deal of difficulty staying focused during lectures. There was a lot of material, and I found myself missing sections because I was on the internet, and then when I was unable to catch up I often gave up. For the second half of this term I am planning on printing notes instead of using my laptop to take away my main source of distractions so I will be better able to focus. When I write I remember information better anyways."*

#### D. Personal Learning Style

##### Level 1

No evidence of recognition is made

##### Level 2

Indicates a preference of one activity from class over another

- "*While studying for this exam, I have learnt that I am someone who needs to understand a concept before I can learn it. I am not someone who can memorize vast amounts of information"*

- "*I preferred the active learning this semester to the didactic lecture. They allowed me to apply what we are learning in class."*

##### Level 3

Recognizes preferred learning style

- "*I find that I am a visual learner. I really like diagrams and slides showing relationships"*

- "*I am an auditory and kinesthetic learner. Repetition of both action and instruction is key for me to grasp a subject"*

##### Level 4

Recognizes a preferred learning style and compares it to teaching style employed in class

- "*I am an extreme multiple-modality learner. The classes with a great lecturer, visual aids, and an object lesson are the ones where I can retain the most information"*

- "*Classes that link topics to clinical situations help me to engage more in the topics and actively participate, as I 'learn-by-doing' better than through being fed information."*

##### Level 5

A concise recognition of learning requirements and ways to facilitate future learning is identified

- "*I learn best through multiple modalities where I can make interconnections. I learned well in class lectures, games, question periods, quizzes and reading the textbook. I find that the more modalities I can involve the more engraved the information becomes. In the future I would like to read the lectures before attending class. This would require will power, but would enable me to ask questions and fully understand what the lecturer is explaining."*

#### E. Connection Between the Course and Past Life Experiences

##### Level 1

No evidence of connections are made

- "*I have learnt how to tell if a tissue growth is malignant or benign." *(Statements such as these throughout the entire paper)

##### Level 2

Limited connections to nonspecific, theoretical aspects of life are discussed

- "*The environmental pathology and obesity lectures were interesting - this affects everyone and will be relevant in every specialty."*

##### Level 3

Connections are recognized but are ineffective

- "*My favourite topic was neoplasia. Despite how fatal this disease can be, it amazes me how it can control its own existence. Every time I learn about metastasis and angiogenesis I am simply in awe of the capabilities of this disease. Now, if only we could find a cure."*

##### Level 4

Connections are made and casually discussed

- "*One of the most intriguing things about this course is it incorporates bits and pieces of information from other courses that I have taken: physiology, histology, anatomy, biochemistry. The course material has taken these subjects and integrated them effectively."*

- "*I found that having a personal experience with cancer in my family increased by interest in the lectures dramatically. I find it much easier to retain information if I'm learning it in hopes of understanding a disease that a friend or a family member suffers from."*

##### Level 5

Connections are explored in great precision and detail

- "*The news in the past few days has been a strong reminder of the importance of pathology in medicine. With the discovery of mistakes made by a pathologist being reported in recent days comes the need to reassess the deaths of patients affected by these mistakes. In class we discussed the various possibilities for errors in a sample's analysis such as pre-analytical, analytical and post-analytical errors. It would be useful to discern where in the analytical process these errors occurred and where improvements could be made."*

#### F. Oneself in Relation to the Situation (Emotions, Values, Beliefs, etc.)

##### Level 1

Employs no 'self' into writing; uses only obvious statements without contextual references

- "*I enjoy topics more as I collect more knowledge about them."*

##### Level2

Generalizations used to explore topics of discussion with 1-2 references to self

- "*The pathology course has opened my eyes and made me feel more comfortable about who I am and how I enjoy learning."*

- "*I see cancer affecting so many people and it can be devastating. It is useful to have some basic knowledge about neoplasia and its diagnosis."*

##### Level3

Both general and personal insight are present, further exploration is required

- "*There is a part of me that refuses to study pathology because I want to deny the fact that cancer even exists. I hate cancer. It is strange for me to be learning about this prevalent disease in the classroom when I know people's lives are challenged by it."*

##### Level 4

Writing reveals a strong connection between the event and the writer accompanied by few general reflections

- "*I am very passionate about sustainable medicine and environmental impact on human and animal health. Things such as air and water quality, pesticides, industrial emissions and GMOs are all interesting to me as eventually I would like to complete a Master's in the environmental impact on health."*

- "*From my personal experiences with various types of cancer, I always figured it was an unbeatable disease. Watching these people pass-on and seeing the effects on the family was devastating. I now realize there is more understood than I believed."*

##### Level 5

Writing reveals a well-developed emotional and personalized relationship to the specific context, enhanced with specific examples

- "*I was very interested in the atherosclerosis and obesity section. I was very intrigued by the statistics and risk factors, mainly because I feel I am at a higher risk for atherosclerosis based on my family history and the bad decisions I made that may be adversely affecting my health. I come from a population that has a high rate of obesity, diabetes, and many other diseases so I feel that as a future health professional, it is one of my responsibilities to try and promote healthy choices. My people are facing an uphill battle when it comes to a lot of social issues but improvements to health of the population as a whole must be addressed. These improvements will be made with proper awareness and education as well as with positive role models in the community like health care professionals with a background in these conditions."*

#### G. Future Applications in a Broadened Spectrum

##### Level 1

No future applications of class materials are discussed

##### Level 2

Future applications of an aspect of the course are mentioned in passing without detail

- "*Learning about pathology is one area which is directly related to some specialties, and indirectly related to others; therefore, I think it is very important to have knowledge about this subject"*

- "*I found this half of pathology interesting because I am interested in oncology as a career."*

##### Level 3

Generalized discussion of possible future implications is discussed is some detail

- "*As a healer I will try to conquer the pathologies we have learned, and I can only do so if I first have an understanding of them"*

##### Level 4

Recognition of few precise important facets of course load expanded to broad future goals

- "*I am beginning to see how integrating pathology is with dentistry. Topics such as neoplasia, amyloid and inflammation will all be important when working within the oral cavity as a future dentist."*

##### Level 5

Explicit details of precise aspects of course load and relate them to specific anticipatory future events

- "*The heart attacks I will see will be due to occlusion that leads to infarction, and the repaired area will consist of a collageneous scar*."

- "*Individuals I will encounter suffering from Alzheimer's disease will have beta-amyloid plaques in the extracellular space of their brains"*

## Pre-publication history

The pre-publication history for this paper can be accessed here:

http://www.biomedcentral.com/1472-6920/11/67/prepub
